# Acute Kidney Injury Post Hip Fracture Surgery: Local Quality Improvement Project and Review of Literature

**DOI:** 10.7759/cureus.75594

**Published:** 2024-12-12

**Authors:** Arsany Metry, Nauman Manzoor, Ahmed Elkilany, Kanishka Wattage, Arnab Sain, Fahad Hussain, Kerollos Khilla, Zain Sohail, Ali Abdulkarim

**Affiliations:** 1 Trauma and Orthopaedics, University Hospitals Sussex National Health Service (NHS) Foundation Trust, Sussex, GBR; 2 General Surgery, University Hospitals Sussex National Health Service (NHS) Foundation Trust, Sussex, GBR; 3 Trauma and Orthopaedics, St. Mary's Hospital, Isle of Wight National Health Service (NHS) Trust, Isle of Wight, GBR

**Keywords:** acute kindey injury, antibiotics in joint replacement, care of elderly, complications of cement, hip fractures

## Abstract

Background: The aim of the study is to identify the potential risk factors for postoperative AKI in hip fracture patients.

Design and methods: Using our local neck of femur (NOF) registration data, patient details were selected using inclusion and exclusion criteria. Electronic records of patients were assessed retrospectively, including blood results, radiological investigations, clinical documentation, and drug charts. The time period was from the start of January 2022 to the end of June 2022. Inclusion Criteria: All patients > 50 years old with NOF fractures underwent operative management from January 2022 to June 2022. Exclusion Criteria: 1. Pathological fractures. 2. Non-operative management. 3. Died directly postoperative.

Results: Two hundred and fifty patients underwent hip fracture surgery at our hospital in 6 months (January 2022-June 2022) (Cemented procedures were 133 [53%], while fixation procedures were 117 [47%]). Female patients were 174 (70%), and male patients were 76. The average age was 83.4 years, and the number of operations done over the weekend (Friday-Sunday) = 123 (49%). The incidence of postoperative AKI was 56 (22.4%). Forty-five of the fifty-six cases were stage one (80.4%), while seven cases (12.5%) were stage 2. The studied risk factors for postoperative AKI were cemented procedures (61% of postoperative AKI incidence), female gender (66%), time from admission to operation (>24 hours = 33%), day of operation (operations done Friday/Saturday/Sunday = 55%), and postoperative antibiotics (71%).

Conclusion: We need strategies to reduce the incidence of postoperative AKI, like AKI alert on laboratory results, IV fluid prescription preoperatively since the arrival of patients to the ED, avoiding/stopping nephrotoxic medications on admission, regular review of postoperative renal function tests and fluid balance, especially in high-risk patients, increase nursing staff and junior doctors on wards over weekends, and we need to review our policy of giving postoperative IV antibiotics.

## Introduction

The term acute kidney injury (AKI) is used to describe a rapid deterioration in renal function occurring over a period of hours to days. This rapid deterioration leads to the accumulation of plasma waste products, such as urea and creatinine [1]. AKI could be a considerable risk factor for the development of chronic kidney disease. Additionally, AKI is recognized for causing damage to distant organs, which subsequently exacerbates morbidity and mortality rates [2].

Post-hip operations AKI is worth investigating as a retrospective study of 644 patients who underwent surgical treatment for hip fractures found a 12.1% incidence of postoperative AKI [3]. Postoperative AKI can increase the length of hospital stay and add more morbidity to the patient [4]. AKI is a temporary but common complication following hip fracture surgery, which can also be predicted if risk factors are adequately observed [5].

As per NICE guidance, one of the cornerstones of the management of AKI is risk assessment [6]. Approaches to identify and monitor patients at risk may improve the outcomes.

The aim of the study is to identify the potential risk factors for postoperative AKI in hip fracture patients. The impact of different preoperative and intraoperative factors was investigated and assessed in relation to postoperative AKI.

## Materials and methods

This is a retrospective study of patients with neck of femur (NOF) fractures who presented to the emergency department of a secondary trauma center. Data was collected using local NOF registration data.

Patients included were any patients more than 50 years old with NOF fractures who underwent operative management from January 2022 to June 2022. At the same time, exclusion criteria included pathological fractures, nonoperative management, and those who died directly postoperatively (within 48 hours). 

Using our local electronic system, preoperative and postoperative blood (including full blood count and renal function tests) were collected. Patients’ demographics were recorded. Preoperative images (including X-rays and CT scans) were reviewed to determine the type of fractures (intra-capsular or extra-capsular). 

Operative notes were reviewed to note the day of the operation, how long the patient had to wait for the operation, and whether cement was used or not.

The electronic prescription system was reviewed to assess if the patient had antibiotics postoperatively or not.

Fixation of fractures was done either using a dynamic hip screw (DHS) device or an intramedullary nailing system, either with a short or long nail.

Replacement procedures were either cemented hemiarthroplasty or total hip replacement, either cemented or uncemented. The cement that was used was Palacos R+G, which is fast-setting bone cement with added gentamicin. 

All of the procedures were done using either lateral or posterior hip approaches.

Outcome measures included: 1) The time the patient had to wait for the operation (< 24 hours / > 24 hours since the arrival to A&E). 2) Giving antibiotics postoperatively. 3) The presence of preoperative AKI. 4) The day of the week that the surgery was done, and patients were divided into 2 groups: one group was done on Friday, Saturday, and Sunday (patients done over the weekend), while the other group was done on Monday, Tuesday, Wednesday, and Thursday (patients done over weekdays). 5) Using of cement intraoperatively.

## Results

Two hundred and fifty patients were included in this study, and regarding the gender distribution, 174 (70%) were females and 76 (30%) were males. The average age was 83.4 years, with the oldest being 105 years and the youngest being 59 years.

**Figure 1:**
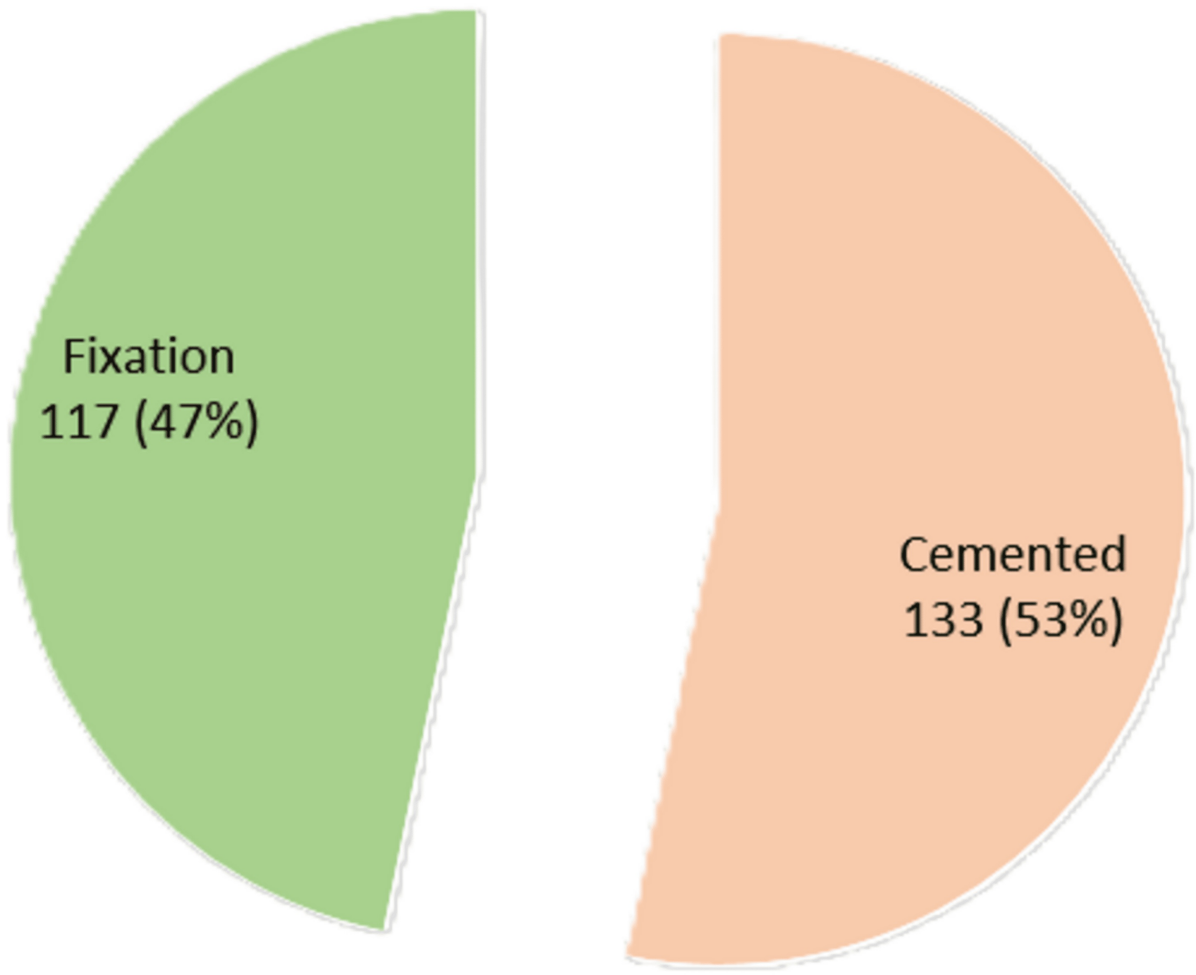
Type of operations

The total number of operations done either using cement or without using cement is illustrated in Figure 1. At more than half of the operations, 133 operations (53%), cement was used.

Patients, who were done over the weekend, were 123 (49% of the total number of operated patients), while the rest were done over the weekdays.

**Figure 2:**
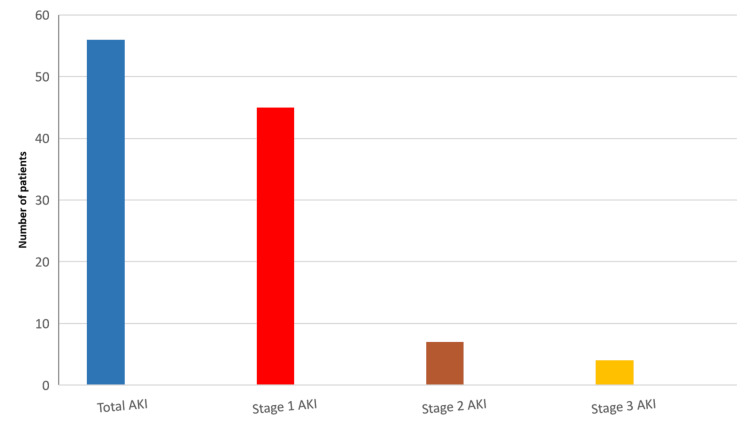
Number of patients at each AKI stage

The incidence of postoperative AKI was 56 (22.4% of the total number of operated patients), and the number of patients at each stage is shown in Figure 2. Forty-five patients (80%) of whom developed post-operative AKI had AKI stage 1. 

Of the 56 patients who developed postoperative AKI, 34 (61%) of them had cemented procedures with a p-value of 0.23. The percentage for each group (cemented and un-cemented) is shown in Table 1.

**Table 1:** Percentage of AKI per each treatment group

Operation	Total	Developed AKI	% AKI
Cemented	133	34	25.6
Fixation	117	22	18.8

The second risk factor for postoperative AKI that was assessed was gender. Thirty-seven (66%) patients who developed postoperative AKI were females, with a p-value of 0.51.

**Figure 3:**
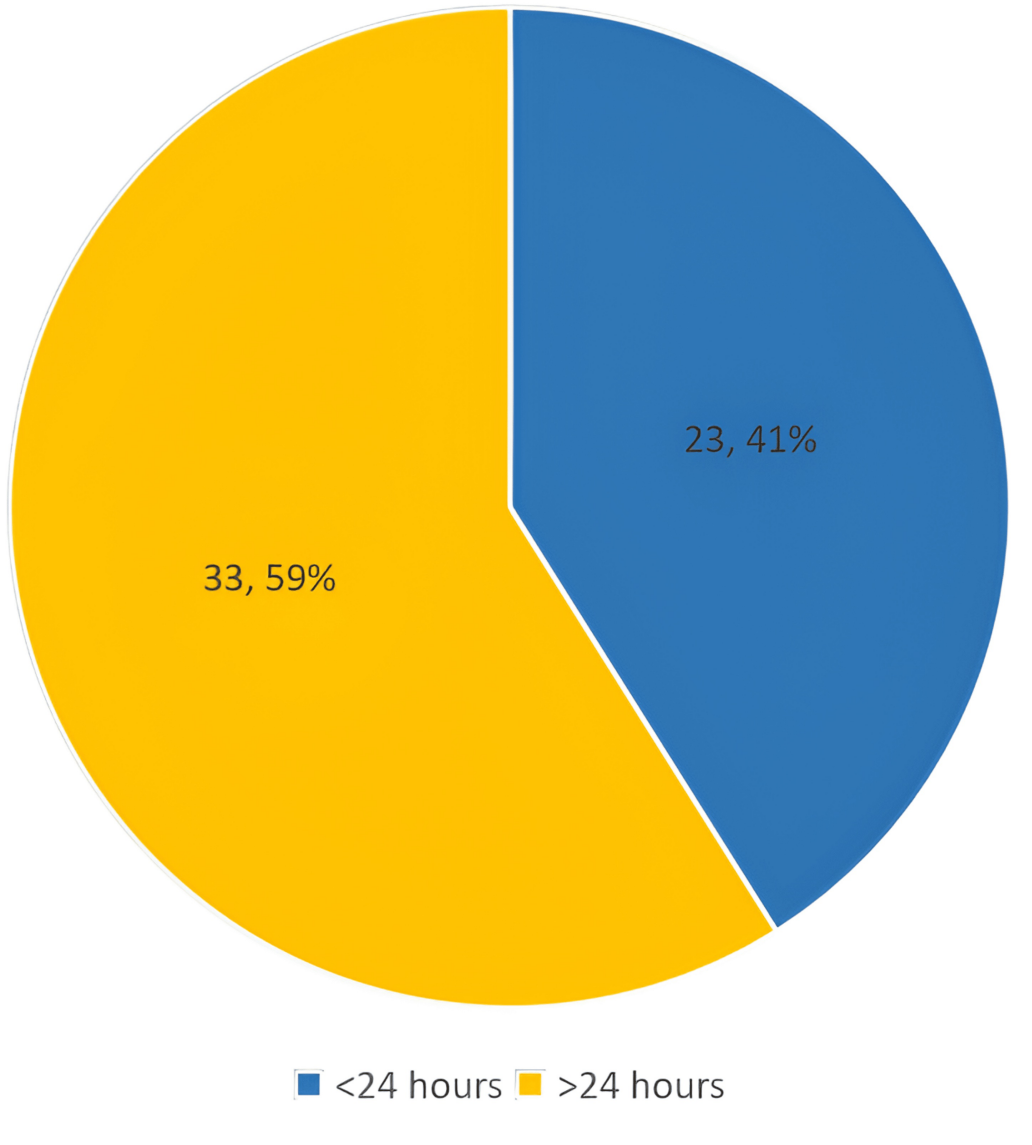
Percentage of AKI in relation to time to go to theatre

Another assessed risk factor was the time patients had to wait to go to the theatre; 33 (58.9%) patients of patients who got postoperative AKI waited more than 24 hours with a P-value of 1. The percentage of AKI among patients who waited more than 24 hours and less than 24 hours is shown in Figure 3.

Ten patients of 250 patients (4%) had AKI preoperatively, and six of them (60%) developed AKI postoperatively with a p-value of 0.01. Of those 10 patients who had preoperative AKI, eight of them (80%) had deterioration of their renal function postoperatively.

**Figure 4:**
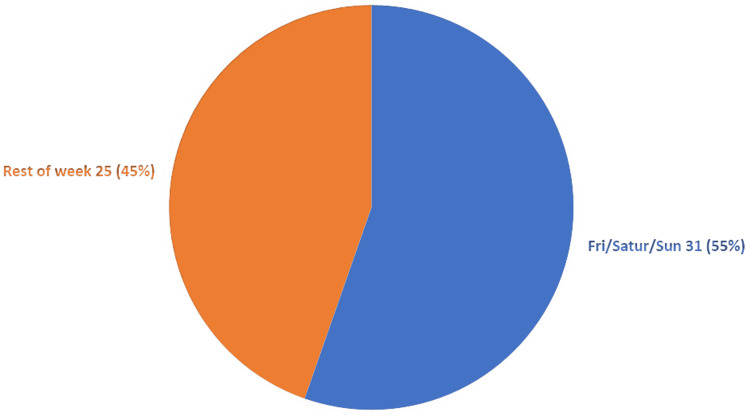
Relation between postoperative AKI and day of surgery

Thirty-one of 56 patients with postoperative AKI had their operations done over the weekend, with a percentage of 55% (Figure 4) and a p-value of 0.36.

Seventy-one percent (71%) of patients who had postoperative AKI had postoperative antibiotics (40 patients) with a p-value of 0.06.

A summary of risk factors and their p-values is shown in Table 2.

**Table 2:** Risk factors and its p-values > 24 H: Time between presentation to emergency department and the operation is more than 24 hours. <24 H: Time between presentation to emergency department and the operation is less than 24 hours. Weekend: Operations done on Friday, Saturday, and Sunday. Weekday: Operations done on Monday, Tuesday, Wednesday, and Thursday.

	Total (n=250)	AKI (n= 56)	Non-AKI (n= 194)	P-Value
* Procedure				0.23
- Cemented	133	34	99	
- Un-cemented	117	22	95	
* Gender				0.51
- Male	76	19	57	
- Female	174	37	137	
* Time to operation				1.00
- > 24 H	147	33	114	
- < 24 H	103	23	80	
* Preoperative AKI				0.01
- Yes	10	6	4	
- No	240	50	190	
* Day of surgery				0.36
- Weekend	123	31	92	
- Weekday	127	25	102	
* Postoperative Antibiotics				0.06
- Yes	151	40	111	
- No	99	16	83	

## Discussion

In our retrospective study of 250 patients who underwent surgical treatment for hip fractures, we found a 22.4% incidence of postoperative AKI. The results of previous studies have shown that the incidence of postoperative AKI after hip fracture ranges from 9.8% to 23.7% [3]. AKI is more common following hip fracture procedures than other postoperative problems, according to several studies, so clinicians need to increase their attention to it [7]. 

Although AKI is temporary, it is a common complication following hip fracture surgery. It typically prolongs the length of hospital stays and increases mortality and morbidity [5]. Kang et al. found postoperative AKI carries a high mortality rate, which may reach 24.0% [8]. Rantalaiho et al. found that AKI can increase mortality risk three-fold during the first three months after surgery for hip fractures in the elderly [9]. That indicates the risk of death is higher with AKI patients in the short term compared with non-AKI patients.

In this study, we observed that preoperative AKI and administering antibiotics postoperatively were significantly correlated with AKI (P < 0.06). Surgeons should be more careful when using postoperative antibiotics, especially after operations using antibiotic-loaded bone cement. Conversely, other factors like using cement, gender, the time the patient had to wait to go to the theatre, and the day of surgery were not statistically correlated with AKI incidents (P > 0.06). Regarding this, our study results are similar to those of some previous studies. Agar et al. showed that pre- and postoperative high urea levels are associated with postoperative AKI [5]. In addition, some studies showed an increase in postoperative renal complications in patients with reduced mobility [10]. The decrease in physical capabilities and diminished requirement for daily activities among elderly individuals may be associated with various comorbid conditions that can hinder their mobility. Consequently, it is essential to consider the aspect of advanced age in patients who are admitted with hip fractures, along with the meticulous management of these individuals.

Although we did not include body mass index (BMI) in the study, previous studies showed that low or high BMI is a risk factor for postoperative AKI in patients undergoing either cardiac or non-cardiac surgeries [11]. Consequently, exploring the mechanisms through which abnormal BMI influences postoperative AKI could aid surgeons in preventing and monitoring the onset of AKI. In relation to this mechanism, several studies have indicated that patients with obesity may possess increased muscle mass, resulting in elevated levels of myoglobin and creatine kinase (CK) release following injury [12]. Obese patients receive less fluid resuscitation during treatment when body weight is considered, and insufficient fluid resuscitation may make rhabdomyolysis-mediated AKI worse [13]. Patients who are underweight may experience hypoalbuminemia and anemia, as well as other forms of malnutrition. This can increase the need for transfusions during the perioperative phase and could be the primary cause of AKI development in underweight patients following surgery [3]. Further research is required to elucidate the mechanism of action to precisely prevent and treat postoperative AKI, as it is still unclear how BMI abnormalities contribute to the development of AKI.

Preoperative clinical variables by themselves do not fully predict postoperative AKI, because some intraoperative or postoperative variables may also be significant risk factors for AKI. The only preoperative factor that was found to be statistically significant was the preoperative AKI.

Some studies showed a correlation between preoperative BNP and postoperative AKI, like Zhao et al. found. Preoperative NT-pro BNP concentrations provided predictive information for AKI in a cohort of patients undergoing non-cardiac surgery [14]. When surgeons discover increased BNP during the postoperative phase, they should take the patient's heart failure into account and be on the lookout for any signs of abnormal renal function, and take the necessary precautions to stop it from happening, like reducing the use of nephrotoxic medications [15].

Limitations

No follow-up after patients’ discharge if they need renal replacement therapy or not. The length of hospital stay was not recorded and compared AKI patients with non-AKI patients, as it was a quality improvement project. Intraoperative blood pressure was not recorded, as it may be one of the primary causes of decreased renal blood flow.

## Conclusions

The etiology of AKI is likely multifactorial in this cohort of patients. Statistically, preoperative AKI and postoperative antibiotics were predictors of AKI after hip fracture surgery. Postoperative AKI can increase the length of hospital stay and may affect short- or long-term mortality. Early management of preoperative AKI could have a great effect on kidney function postoperatively.
